# Plasma Cell-Free DNA Levels Are Elevated in Acute Puumala Hantavirus Infection

**DOI:** 10.1371/journal.pone.0031455

**Published:** 2012-02-07

**Authors:** Tuula K. Outinen, Taru Kuparinen, Juulia Jylhävä, Sonja Leppänen, Jukka Mustonen, Satu Mäkelä, Ilkka Pörsti, Jaana Syrjänen, Antti Vaheri, Mikko Hurme

**Affiliations:** 1 Department of Internal Medicine, Tampere University Hospital, Tampere, Finland; 2 School of Medicine, University of Tampere, Tampere, Finland; 3 Department of Virology, Infection Biology Research Program, Haartman Institute, University of Helsinki, Helsinki, Finland; 4 Fimlab Laboratories, Tampere, Finland; University of Calgary & ProvLab Alberta, Canada

## Abstract

**Introduction:**

Puumala hantavirus (PUUV) causes a hemorrhagic fever with renal syndrome called nephropathia epidemica (NE). The aim of the present study was to evaluate plasma cell-free DNA (cf-DNA) levels and urinary cf-DNA excretion in acute NE as well as their associations with the severity of the disease.

**Methods:**

Total plasma cf-DNA was quantified directly in plasma of 61 patients and urine of 20 patients with acute NE. We also carried out a qualitative high-sensitivity lab-on-a-chip DNA assay in 20 patients to elucidate the appearance of cf-DNA in plasma and urine.

**Results:**

The maximum plasma cf-DNA values taken during acute NE were significantly higher than the control values taken after the hospitalization period (median 1.33 µg/ml, range 0.94–3.29 µg/ml *vs.* median 0.77 µg/ml, range 0.55–0.99 µg/ml, *P*<0.001). The maximum plasma cf-DNA levels correlated positively with maximum blood leukocyte count (*r* = 0.388, *P* = 0.002) and the length of hospital stay (*r* = 0.376, *P* = 0.003), and inversely with minimum blood platelet count (*r* = −0.297, *P* = 0.020). Qualitative analysis of plasma cf-DNA revealed that in most of the patients cf-DNA displayed a low-molecular weight appearance, corresponding to the size of apoptotic DNA (150–200 bp). The visually graded maximum cf-DNA band intensity correlated positively with the maximum quantity of total plasma cf-DNA (*r* = 0.513, *P* = 0.021). Maximum urinary excretion of cf-DNA in turn was not markedly increased during the acute phase of NE and did not correlate with any of the variables reflecting severity of the disease or with the maximum plasma cf-DNA level.

**Conclusions:**

The plasma levels of cf-DNA are elevated during acute PUUV infection and correlate with the apoptotic cf-DNA-band intensity. The plasma cf-DNA concentration correlates with some variables reflecting the severity of the disease. The urinary excretion of cf-DNA does not reflect the degree of inflammation in the kidney.

## Introduction

Puumala hantavirus (PUUV) is a rodent-borne zoonotic virus carried by the bank vole, *Myodes glareolus* [1]. PUUV causes a mild hemorrhagic fever with renal syndrome called nephropathia epidemica (NE) [2]. Numerous hantaviruses cause hemorrhagic fever with renal syndrome in Eurasia and hantavirus cardiopulmonary syndrome in the Americas [2]. In Finland, 1000–3000 serological NE diagnoses are made annually (http://www3.ktl.fi).

The severity of PUUV infection varies from subclinical disease to rare fatal cases [3]. Host genetics have been shown to influence the clinical picture [4]. After an incubation period of 2–6 weeks, the disease begins with sudden high fever, headache, back and abdominal pains, nausea and visual disturbances [5]–[6]. Signs of renal involvement are proteinuria and hematuria, as well as oliguria followed by polyuria [5]–[6]. Five per cent of hospitalized patients need transient hemodialysis treatment [2]. Typical laboratory findings during the acute phase are leukocytosis, thrombocytopenia, anemia, and elevation of plasma C-reactive protein (CRP) and creatinine levels [6]. Radiological pulmonary manifestations have been detected in about one-third of NE patients [7]–[9]. In addition, over half of patients have abnormal cardiac findings [10].

The pathogenesis of NE is not completely understood. An important feature in hantaviral infections is capillary leakage due to increased capillary permeability. The mechanisms behind this phenomenon are unclear, but complement activation may be involved [11]–[13]. Immunological responses rather than direct cytotoxicity of the virus have been suggested to be important in the pathogenesis of hantaviral infections. One reason for this hypothesis is that hantaviruses are considered noncytopathic [14]–[15], although under certain conditions Tula hantavirus induces apoptosis in cultured cells [16]–[17]. Increased cytokine levels have been found in the plasma, urine and tissues in patients with hantaviral infection [18]–[21], and in our previous study, high interleukin (IL)-6 levels also associated with a clinically severe course of NE [22]. In addition, high levels of indoleamine 2,3 dioxygenase (IDO) and pentraxin-3 (PTX3), elements of the innate immunity, were associated with a clinically severe NE in our previous studies [23]–[24]. Although no direct viral cytopathy has been detected, increased levels of serum lactate dehydrogenase, aspartate aminotransferase, and alanine aminotransferase have been observed in patients with hantaviral infection, indicating that the cellular membrane integrity is disturbed [25]. A recent study in PUUV-infected patients showed that epithelial cell apoptosis is induced during acute infection and suggests that the tissue damage is due to immune reaction [26].

Circulating cell-free DNA (cf-DNA) has recently been studied in various acute and chronic clinical disorders. Elevated levels of cf-DNA have been reported in cancer, autoimmune diseases, stroke, myocardial infarction, trauma patients and sepsis [27]–[34]. It has also been proposed that cf-DNA could be used as a predictor of outcome or disease severity in these conditions [35]. Detectable levels of cf-DNA are present also in the plasma of healthy individuals, but the concentrations are low [36]. However, a recent study showed cf-DNA concentrations to be elevated in elderly women [37]. The current view is that in clinical conditions cf-DNA originates from apoptotic or necrotic cells and therefore reflects the amount of cellular damage [27].

Studies on plasma cf-DNA in viral infections are scarce and urine levels of cf-DNA have not previously been studied in infectious diseases. In the present study, our aim was to assess cf-DNA plasma levels and urine excretion in patients with acute PUUV infection. We aimed to evaluate the associations of plasma cf-DNA levels with the clinical severity of the disease. We also wanted to assess whether the excretion of cf-DNA is associated with the severity of the infection or renal insufficiency. In addition to measuring total cf-DNA levels directly in plasma and urine, we carried out a qualitative high-sensitivity lab-on-a-chip DNA assay to elucidate the appearance of cf-DNA in plasma and urine during the course of the infection.

## Materials and Methods

### Patients

The study cohort consisted of 61 prospectively collected consecutive adult patients with acute NE. The diagnosis of acute PUUV infection was serologically confirmed in all cases [38]. The patients were treated at the Tampere University Hospital (Tampere, Finland) from September 2000 to December 2004. The median patient age was 46 (range 22–77) years, and 44 (72%) were males. We have previously studied IL-6 and CRP in 118 NE patients [22], as well as IDO in 102 NE patients [23]. Forty-eight of the patients in the present study were also included in the IL-6 and CRP study, and all 61 patients in the present study were also included in the IDO study. We have also previously studied complement activation as well as PTX3 in NE in the same cohort of 61 patients [13], [24]. Furthermore, 19 of the patients in the present study were also included in studies examining the activation of coagulation and fibrinolysis as well as platelet ligands and endothelial involvement in NE [39]–[40].

The following diseases before NE were prevalent in 24 (39%) patients: essential hypertension in eight; dyslipidemia in six; atrial fibrillation in three; coronary artery disease, bronchial asthma, hypothyreosis, chronic inflammatory bowel disease, and hyperplasia of the prostate in two patients each; diabetes mellitus, sick sinus syndrome treated with pacemaker, Sjögren's syndrome, mitral valve disease, epilepsy, fibromyalgia, sarcoidosis, multiple sclerosis, operated atrial septal defect, and operated melanoma were present in one patient each.

All subjects gave an informed consent before participation and the study was approved by the Ethics Committee of Tampere University Hospital.

### Study protocol

All 61 patients were examined during the acute phase of NE. A detailed past and current medical history was obtained, and a careful physical examination was performed. Blood samples were collected between 7:30–9:30 in the morning for up to five consecutive days after hospitalization. They were used for the analysis of plasma cf-DNA, PTX3, IL-6 (from 48 patients), CRP, and creatinine, as well as serum kynurenine (Kyn) and tryptophan (Trp) levels, and also for the blood cell counts. Other blood samples were taken according to the clinical needs of the patient.

The nightly urine collection was performed during two consecutive nights after hospitalization. The nightly collection period was set from the time of the last voiding at bedtime until the last voiding on rising. After completion, volume was measured and timing was recorded for the collection period.

The highest and the lowest values of the various variables measured during hospitalization for each patient were designated as the maximum and minimum values.

One to three chest radiographs were obtained from 38 patients (62%).

Fifty-three (87%) of the 61 patients were also studied at the out-patient clinic four weeks after the hospital period. The plasma and urine samples taken four weeks after the hospital treatment were regarded and assessed as control/recovery samples.

## Methods

### Quantification analyses of plasma and urine cf-DNA

The cf-DNA analyses were performed afterwards from frozen samples stored at −70°C. The amount of total cf-DNA was determined directly in plasma and urine without any DNA purification step, using the Quant-iT™ high-sensitivity DNA assay kit and a Qubit® fluorometer (Invitrogen, Carlsbad, CA, USA) following the manufacturer's instructions. Plasma samples were analysed in duplicate and the mean of the two values was used as the final value. The assessed intra-day variation coefficients at the mean plasma cf-DNA levels of 0.673 µg/ml, 0.876 µg/ml and 1.59 µg/ml were 4.2%, 1.0% and 4.1%, respectively. The corresponding inter-day variation coefficients were 5.5%, 4.3% and 6.6%. Total cf-DNA in urine was measured in 20/61 patients with quadruple measurements in which the mean of the four values was used as the final value. The assessed intra-day variation coefficients at the mean urine levels of 0.307 µg/ml, 0.769 µg/ml and 1.13 µg/ml were 5.0%, 4.8% and 3.5%, respectively. The corresponding inter-day variation coefficients were 10.0%, 6.5% and 10.2%. Timed overnight urinary excretion of cf-DNA was calculated as follows: (concentration×total volume)/(time span).

### Extraction and qualitative analysis of cf-DNA in plasma and urine

Qualitative analysis of plasma and urine cf-DNA was performed for randomly selected 10 patients with and without renal insufficiency (defined as maximum plasma creatinine >370 µmol/l and maximum plasma creatinine <125 µmol/l, respectively). Plasma and urine cf-DNA was extracted using the NucleoSpin® Plasma XS Kit (MACHEREY-NAGEL GmbH & Co., Düren, Germany), designed for isolation of low-molecular-weight (50–1000 bp) cf-DNA. Cf-DNA isolation was performed according to the manufacturer's instructions following the high-sensitivity protocol. Extracted cf-DNA samples were analyzed with the High Sensitivity DNA assay kit and an Agilent 2100 Bioanalyzer equipped with Expert 2100 software according to the manufacturer's instructions (Agilent Technologies Inc., Santa Clara, CA). Agilent 2100 Bioanalyzer uses a lab-on-a-chip technology to perform gel electrophoresis; nucleic acids are separated analogously to a capillary electrophoresis and normalized to a ladder and two DNA markers, after which the software automatically calculates the size of each band. For each plasma sample, the appearance and intensity of low-molecular weight cf-DNA was estimated visually and graded as follows: 1 = no visible cf-DNA or extremely weak band intensity, 2 = intermediate band intensity, 3 = strong band intensity. The researcher responsible for analysing and grading the cf-DNA samples was blinded for the clinical data of the patients. The appearance of cf-DNA in urine was analyzed descriptively.

Plasma CRP and creatinine levels were analyzed using Cobas Integra analyzer (F. Hoffman-La Roche Ltd, Basel, Switzerland). Blood cell count was completed by hematological cell counters by Bayer. Plasma IL-6 concentrations were determined as previously described [21]. IDO level can be measured by determining the ratio of Kyn to Trp in serum [41] by reverse-phase high performance liquid chromatography (HPLC) as previously described [42]. The Kyn/Trp ratio was calculated by relating concentrations of Kyn to Trp. Plasma PTX3 determinations were performed by using a commercially available human pentraxin-3 immunoassay (Quantikine, R&D Systems, Inc., Minneapolis, MN), following the manufacturer's instructions. Plasma IL-6 and PTX3 as well as serum Kyn and Trp concentrations were measured afterwards from frozen samples. All laboratory variables mentioned above were determined at the Laboratory Center of Pirkanmaa Hospital District.

### Statistical Analyses

In order to describe the data, medians and ranges were given for continuous variables and numbers and percentages for categorical variables. Groups were compared using the Mann-Whitney *U-* test. Categorical data were analyzed by the *x* ^2^ test or the Fisher's exact test, as appropriate. Correlations were calculated by the Spearman's rank correlation test. Wilcoxon's test was used to compare two related samples. All tests were two-sided, and statistically significant *P*-values are given. All analyses were made with the SPSS (version 18) statistical software package.

## Results

The clinical characteristics of the 61 patients are shown in Table 1 and the laboratory variables in Table 2. None of the patients was in clinical shock at the time of admission. Four patients (7%) needed hemodialysis treatment during the hospital stay. Eleven patients had pathologic findings in chest radiograph, i.e. 29% of the 38 patients with radiograph performed. No deaths occurred.

**Table 1:** Clinical data for 61 patients with Puumala hantavirus infection.

	Median	Range
Age (years)	46	22–77
BMI (kg/m^2^)	25.1	19.8–35.7
Duration of fever before hospital admission (days)	4	1–15
Duration of fever (days)	6	2–19
Length of hospital stay (days)	6	2–15
Urinary output min (ml/day)	1600	50–4940

BMI = body mass index, min = minimum, max = maximum

**Table 2:** Laboratory data for 61 patients with Puumala hantavirus infection.

	Median	Range
Creatinine max (µmol/l)	175	65–1285
Platelets min (10E9/L)	68	9–238
Hematocrit min	0.36	0.25–0.43
Leukocytes max (10E9/L)	9.9	3.9–31.2
CRP max (mg/l)	69.2	16.7–269.2
IL-6 max (pg/ml) (n = 48)	11.5	1.3–96.6
IDO max (µmol/mmol)	212.3	46.6–3679.2

Min = minimum, Max = maximum, CRP = plasma C-reactive protein, IL-6 = plasma inteleukin-6, IDO = serum kynurenine/tryptophan ratio.

The maximum total plasma cf-DNA values taken during acute NE were significantly higher than the control values taken after the hospitalization period (median 1.33 µg/ml, range 0.94–3.29 µg/ml *vs.* median 0.77 µg/ml, range 0.55–0.99 µg/ml, *P*<0.001). Maximum urinary excretion of cf-DNA was not increased during the acute phase of NE when compared with the control values after the hospitalization (median 0.68 µg/min, range 0.34–1.38 µg/min *vs.* median 0.62 µg/min, range 0.19–1.15 µg/ml, *P* = 0.43). The control values were taken median 41 (range 18–83) days after the onset of fever.

The maximum plasma cf-DNA levels correlated positively with maximum blood leukocyte count (*r* = 0.388, *P* = 0.002), maximum plasma PTX3 levels (*r* = 0.513, *P*<0.001), and the length of hospital stay (*r* = 0.376, *P* = 0.003). There was also an inverse correlation between maximum plasma cf-DNA levels and minimum blood platelet count (*r* = −0.297, *P* = 0.020). The maximum plasma cf-DNA levels did not correlate with maximum plasma creatinine levels or minimum urinary output (*r* = 0.101, *P* = 0.436 and *r* = −0.063, *P* = 0.636; respectively). Neither did the maximum cf-DNA levels correlate with minimum hematocrit, maximum plasma CRP, IL-6 or serum IDO levels (*r* = −0.120, *P* = 0.359; *r* = −0.015, *P* = 0.907; *r* = 0.202, *P* = 0.168 and *r* = 0.228, *P* = 0.077; respectively). There was no correlation between plasma cf-DNA and age (*r* = 0.093, *P* = 0.477).

There was no significant difference in maximum plasma cf-DNA levels between patients with normal or pathologic chest radiograph, or between patients who needed hemodialysis treatment and those who managed without hemodialysis. Also, the plasma cf-DNA levels did not differ between men and women (data not shown).

The maximum urinary excretion of cf-DNA did not correlate with any of the variables reflecting severity of the disease, and it did not correlate with age or maximum plasma cf-DNA, either (data not shown).

**Figure 1:**
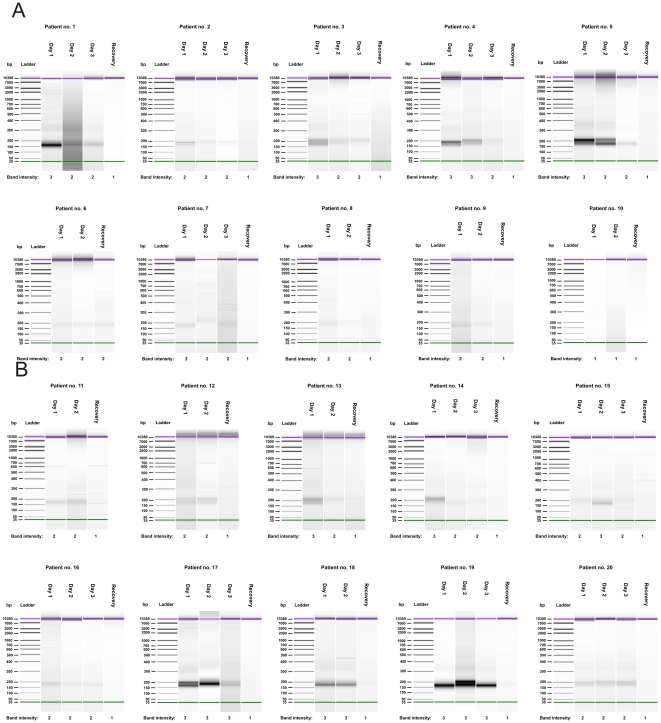
Qualitative analysis of plasma cf-DNA in 10 patients with maximum plasma creatinine >370 µmol/l (A) and 10 patients with maximum plasma creatinine <125 µmol/l (B) after NucleoSpin® Plasma XS kit extraction. Analyses were performed with Agilent's High Sensitivity Lab-on-a-chip DNA assay. Green lines indicate the low weight (35 base pairs (bp)) DNA marker and purple lines the high weight (10 380 bp) DNA marker. The intensity of low-molecular weight cf-DNA band was graded as follows: 1 = no visible cf-DNA or weak band intensity, 2 = intermediate band intensity, 3 = strong band intensity.

Qualitative analysis of plasma cf-DNA revealed that during the acute phase of the disease, in most patients cf-DNA displayed a low-molecular weight appearance, corresponding to the size of apoptotic DNA fragments (150–200 bp) (Figures 1A and 1B). This phenomenon was observed in patients with and without renal insufficiency. The visually graded maximum cf-DNA band intensity correlated positively with the maximum quantity of total plasma cf-DNA (*r* = 0.513, *P* = 0.021). However, the maximum cf-DNA band intensity did not correlate with any of the clinical or laboratory parameters. In control samples, which were taken four weeks after the hospital period, the low-molecular weight cf-DNA band was either completely absent or markedly weakened in all patients (Figures 1A and 1B).

**Figure 2:**
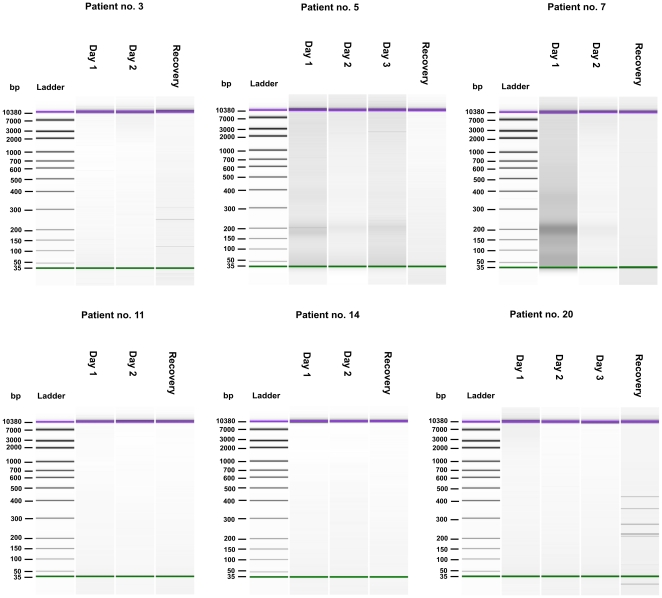
Qualitative analysis of urine cf-DNA after NucleoSpin® Plasma XS kit extraction. Analyses were performed with Agilent's High Sensitivity Lab-on-a-chip DNA assay. Green lines indicate the low weight (35 base pairs (bp)) DNA marker and purple lines the high weight (10 380 bp) DNA marker. During the acute phase of the disease low-molecular weight (150–200 bp) pattern of cf-DNA was detected only in patients no 5 and 7, while patients no 3 and 20 had random-sized cf-DNA fragments in their control urine samples. Data from patients no 11 and 14 are depicted as examples of the 16 subjects who had no findings in the urinary cf-DNA fragment analyses.

Qualitative analysis of urine-cf-DNA revealed that in contrast to plasma, no distinguishable low-molecular weight (150–200 bp) pattern of cf-DNA was detected during the acute phase of the disease, with the exception of two patients with renal insufficiency (patients no 5 and 7 with maximum plasma creatinine >370 µmol/l) (Figure 2). In addition, two patients (no 3 and 20) had random-sized cf-DNA fragments in their control urine samples (Figure 2). The tracings of patients no 11 and 14 are shown as examples of the 16 patients who had no findings in the urinary cf-DNA fragment analyses (Figure 2).

## Discussion

The data presented here show that plasma cf-DNA levels are elevated during acute PUUV infection. The cf-DNA values measured during the acute phase were markedly higher than the values measured after the hospitalization. Previously, in bacteremia patients the median maximum cf-DNA levels were 2.03 µg/ml in nonsurvivors and 1.26 µg/ml in survivors [34]. In the present study, the median maximum plasma cf-DNA levels (1.33 µg/ml) were close the level of survived bacteremia patients. In the study presented here, the total plasma cf-DNA levels did not correlate with the severity of renal insufficiency. However, there was an inverse correlation between plasma cf-DNA level and blood platelet count. In addition, plasma cf-DNA levels correlated positively with blood leukocyte count and the length of hospital stay, which is probably one of the best variables reflecting the overall severity of the disease as all patients fully recovered. Plasma cf-DNA levels also correlated with plasma PTX3 levels. This is logical as PTX3 contributes to the opsonization and clearance of apoptotic or necrotic cells [43], which are regarded as the origin of cf-DNA. In contrast, urine maximum cf-DNA excretion did not correlate with any clinical or laboratory parameters or maximum plasma cf-DNA.

Qualitative analysis of plasma cf-DNA revealed that, during the acute phase of infection, cf-DNA displayed a predominance of low-molecular weight and apoptotic (150–200 bp) appearance, whereas after recovery, such cf-DNA pattern was not observed in any of the patients. It is thus likely that the detected low-molecular weight cf-DNA originated from apoptotic cells in the course of the acute phase of the disease. In fact, results from a recent study in PUUV infected patients suggest that acute hantavirus infection is associated with immune reaction-induced renal tissue damage [26]. Moreover, the observed correlation between maximum total plasma cf-DNA concentration and the low-molecular weight cf-DNA band intensity supports the hypothesis that the increase in plasma cf-DNA is due to apoptosis. Notably, NE is a general infection and thus the low-molecular weight DNA fragments could be derived from a variety of affected tissues. Similar results in qualitative cf-DNA pattern have recently been observed in bacteremia patients [34]. However, in the current study, the plasma cf-DNA band intensity was not associated with the level of renal function or clinical picture of the disease. We did not detect any qualitative patterns in urine cf-DNA that could be attributed to the disease severity, either. Nevertheless, two patients with renal insufficiency had a clear low-molecular weight cf-DNA band in the urine during the acute phase of the disease, potentially indicating increased apoptosis in the renal system.

The observation that the correlates for maximal total urinary cf-DNA excretion were different from those of the maximum plasma cf-DNA concentration suggest that the amount of cf-DNA in urine may not be clinically relevant and the excretion of cf-DNA does not reflect the degree of inflammation in the kidneys in acute NE. Our findings are corroborated by previous results which have demonstrated that in hematopoietic stem cell transplant patients, the quantity of donor-derived cf-DNA in urine does not correlate with that of plasma and that the predominant cf-DNA fragment size differs between plasma and urine [44]–[45]. A similar phenomenon regarding the DNA fragment size discrepancy between plasma and urine cf-DNA has also been observed in pregnant women [46]. In fact, urinary cf-DNA is likely to consist of a heterogeneous mixture of cf-DNA fragments originating from dying cells in the renal system and from the pool of plasma circulating cf-DNA. The former is supported by the observation that patients with urinary tract infection have elevated urine cf-DNA levels [44], whereas the latter has been demonstrated in colorectal cancer patients who displayed tumor-derived mutated *K-ras* sequences in the transrenal urine cf-DNA [47]–[48]. Likewise, Y-chromosomal DNA sequences have been detected in the urine of pregnant women carrying male fetuses [44]. Based on animal studies, however, it has been estimated that only 0.5–2% of the circulating DNA passes through the kidneys and is excreted into the urine in a polymeric form [44], [49]. The exact mechanism by which cf-DNA crosses the glomerular basement membrane is currently unknown, yet the maximum urinary excretion of cf-DNA is anticipated to be influenced by renal function although our current results do not seem to support this hypothesis.

Previously cf-DNA has been investigated in a variety of clinical conditions. However, studies concerning infections other than sepsis are scarce. In septic patients, there are studies showing elevated cf-DNA levels that also predict outcome [32], [34], [50]–[51]. In trauma patients, cf-DNA predicted inflammatory second hit and sepsis [52]. In febrile patients, cf-DNA showed prognostic value in assessing the probability and severity of infection and sepsis [53]. In viral infections, elevated levels of cf-DNA have been found in patients with occult hepatitis B [54].

In conclusion, the plasma levels of cf-DNA are elevated during acute PUUV infection and correlate with the apoptotic band intensity. The total plasma cf-DNA concentration correlates with some variables reflecting the severity of the disease. The urinary excretion of cf-DNA does not reflect the degree of inflammation in the kidney.
